# Aged garlic has more potent antiglycation and antioxidant properties compared to fresh garlic extract *in vitro*

**DOI:** 10.1038/srep39613

**Published:** 2017-01-04

**Authors:** Abdulhakim Elosta, Mark Slevin, Khalid Rahman, Nessar Ahmed

**Affiliations:** 1 School of Healthcare Science, Manchester Metropolitan University, Manchester M1 5GD, United Kingdom; 2 School of Pharmacy and Biomolecular Sciences, Liverpool John Moores University, Liverpool L3 3AF, United Kingdom

## Abstract

Protein glycation involves formation of early (Amadori) and late advanced glycation endproducts (AGEs) together with free radicals via autoxidation of glucose and Amadori products. Glycation and increased free radical activity underlie the pathogenesis of diabetic complications. This study investigated whether aged garlic has more potent antiglycation and antioxidant properties compared to fresh garlic extract *in vitro* in a cell-free system. Proteins were glycated by incubation with sugars (glucose, methylglyoxal or ribose) ±5–15 mg/mL of aged and fresh garlic extracts. Advanced glycation endproducts were measured using SDS-PAGE gels and by ELISA whereas Amadori products were assessed by the fructosamine method. Colorimetric methods were used to assess antioxidant activity, free radical scavenging capacity, protein-bound carbonyl groups, thiol groups and metal chelation activities in addition to phenolic, total flavonoid and flavonol content of aged and fresh garlic extracts. Aged garlic inhibited AGEs by 56.4% compared to 33.5% for an equivalent concentration of fresh garlic extract. Similarly, aged garlic had a higher total phenolic content (129 ± 1.8 mg/g) compared to fresh garlic extract (56 ± 1.2 mg/g). Aged garlic has more potent antiglycation and antioxidant properties compared to fresh garlic extract and is more suitable for use in future *in vivo* studies.

Chronic hyperglycaemia of diabetes mellitus underlies the development of long-term complications afflicting the eyes, nerves, blood vessels, kidneys and skin of affected individuals. Hyperglycaemia increases protein glycation, which is the non-enzymatic reaction between carbonyl groups from reducing sugars and protein amino groups forming an unstable Schiff base that rearranges to a more stable Amadori product. Once formed, the Amadori products undergo further reactions involving dicarbonyl intermediates such as 3-deoxyglucosones and ultimately forming fluorescent and cross-linked structures called advanced glycation endproducts (AGEs). Accumulation of tissue AGEs have been implicated in the development of diabetic complications^1^,^2^. Protein glycation is accompanied by free radical production from autoxidation of glucose (autoxidative glycation) and oxidation of glycated proteins (glycoxidation) to from AGEs^2^. Free radicals are capable of damaging biomolecules and this oxidative stress contributes towards the pathogenesis of diabetic complications^2^.

Despite advances in treatment, the long-term complications of diabetes are a major cause of concern. Thus, inhibition of AGE-mediated tissue damage and oxidative stress may offer therapeutic potential for preventing or delaying onset or progression of diabetic complications^1^. Various antiglycation compounds have been studied and include aminoguanidine, a drug found to be effective both *in vitro* and *in vivo*^1^. Other compounds include AGE cross-link breakers such as N-phenacylthiazolium bromide have been proposed as agents for reversing protein cross-linking caused by AGEs^3^. Furthermore, there is considerable interest in compounds capable of blocking interaction between AGEs and their receptor called receptor for AGEs (RAGE)^4^. Although many compounds have been studied, none has been approved for clinical use. Whilst the search for new synthetic antiglycation agents continues, little attention has been devoted to antiglycation agents from natural sources. Various studies have indicated that dietary supplementation with nutrients possessing both antiglycation and antioxidant properties may be a safe and simple complement to traditional therapies aimed at preventing diabetic complications^5^.

Garlic (*Allium sativum*) has been used as a flavouring agent, functional food and in folklore medicine. Indeed, its medicinal properties have been used to treat a wide variety of diseases since ancient times^6^. Garlic is a well-established prophylactic and therapeutic medicinal agent, which has been investigated for health benefits, resulting in more than 3,000 publications from all over the world^7^. Garlic is a rich source of water- and lipid-soluble organosulphur compounds, but the constituents responsible for the health benefits of garlic may vary in type and concentration, depending on different processing, preparations and soil conditions^8^. Garlic is available in different formulations; one of which is aged garlic extract and manufactured by soaking sliced raw garlic in 15–20% aqueous ethanol for up to 20-months at room temperature. The extract is then filtered and concentrated under reduced pressure at low temperature. During this ageing, the odorous and harsh irritating compounds in garlic are converted to odourless, non-irritating, safe sulphur compounds^9^. This process causes loss of allicin and increases activity of other water-soluble organosulphur compounds, such as S-allyl cysteine (SAC), S-allyl mercaptocysteine (SAMC), allixin and selenium, all of which are antioxidants^8^,^10^. Another antioxidant present in aged garlic extract is N-fructosyl arginine, which is not present in raw or heat-treated garlic preparations^11^. The aim of this study was to investigate and compare the abilities of aged and fresh garlic extracts to inhibit formation of AGEs. Furthermore, the antioxidant activity of aged garlic extract was also investigated and compared with that of fresh garlic extract.

## Results

### Effects of aged and fresh garlic extracts on cross-linked AGEs

**Figure 1:**
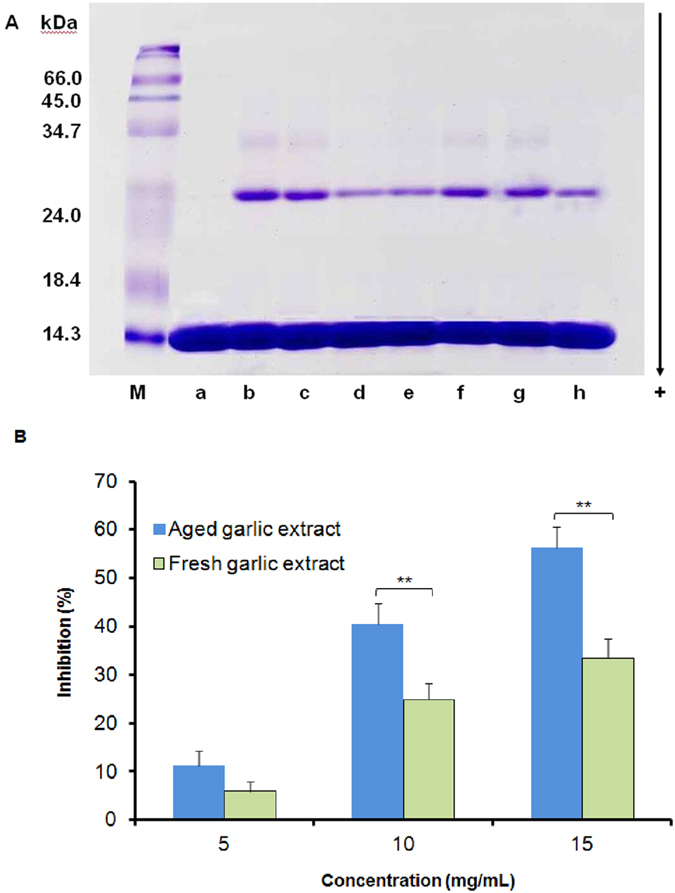
(**A**) SDS-PAGE gel showing the effects of aged and fresh garlic extracts on formation of crosslinked AGEs formed by glycation of lysozyme (10 mg/mL) by 0.5 M glucose in 0.1 M phosphate buffer, pH 7.4 for 4 weeks at 37 °C. Unmodified lysozyme (lane a), glycated lysozyme (lane b) or lysozyme glycated in the presence of 5 (lane c), 10 (lane d) or 15 mg/mL (lane e) of aged garlic extract or 5 (lane f), 10 (lane g) or 15 mg/mL (lane h) of fresh garlic extract. (**B**) Percentage inhibition of crosslinked AGEs. Each value represents the mean ± SD (n = 3). Statistical significance was defined as **P < 0.01 compared to aged garlic extract.

Glycation of lysozyme by glucose produces crosslinked AGEs that present as dimers with an approximate molecular weight of 28 kDa. Glycated lysozyme (Fig. 1A, lane b) showed reduced electrophoretic mobility for the lysozyme monomer and the presence of dimers formed via AGE crosslinks compared to native lysozyme (Fig. 1A, lane a). Aged garlic (Fig. 1A, lanes c–e) and fresh garlic extracts (Fig. 1A, lanes f–h) inhibited AGE-induced dimerization of lysozyme in a dose-dependent manner. The mean values for aged garlic extract at 5, 10 and 15 mg/mL concentrations were 11.2%, 40.4% and 56.4%, compared to 5.9%, 24.9% and 33.5% for fresh garlic extract, respectively. Aged garlic extract showed a significantly (*P* < 0.01) greater inhibitory effect compared to fresh garlic extract at higher concentrations only (10–15 mg/mL) (Fig. 1B).

### Amadorin activity of aged and fresh garlic extracts

**Figure 2:**
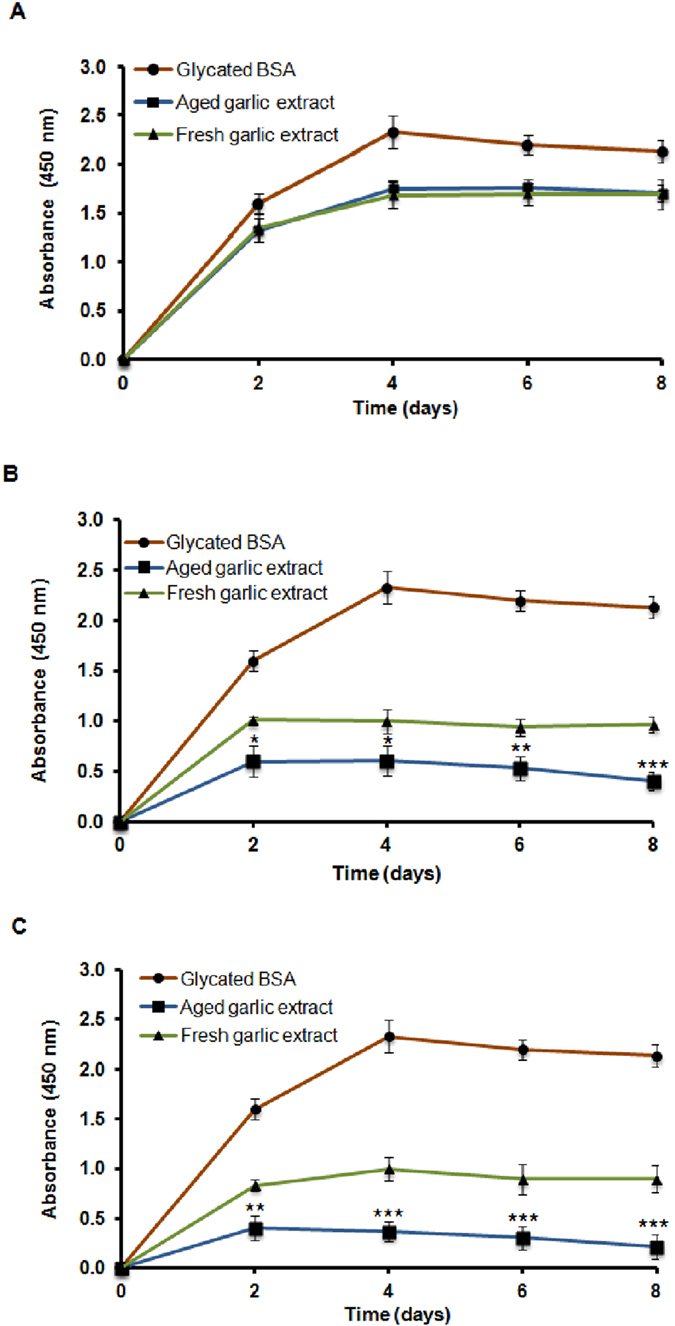
Effect of (**A**) 5 mg/ml, (**B**) 10 mg/ml and (**C**) 15 mg/ml of aged and fresh garlic extracts on post-Amadori AGE formation measured using ELISA at different time points. Results are presented as mean ± SD (n = 3). At each time interval, statistical significance was defined as *P < 0.05, **P < 0.01, ***P < 0.001 compared to fresh garlic extract.

Reincubation of dialysed Amadori-rich bovine serum albumin (BSA) following glycation with ribose results in the formation of AGEs as shown in Fig. 2. The kinetics of post-Amadori AGE formation was rapid over 2 days in the absence of any inhibitors. Both aged and fresh garlic extracts inhibited these post-Amadori reactions with aged garlic extract being more effective at all concentrations tested compared to glycated BSA. At low concentrations (5 mg/mL), aged garlic extract showed no significant inhibition of post-Amadori AGE formation compared to fresh garlic extract (Fig. 2A). From the data in Fig. 2B and C, it is apparent that aged garlic extract at higher concentrations (10–15 mg/mL) showed significant (*P* < 0.001) inhibition of post-Amadori AGE formation after 8 days compared to fresh garlic extract.

### Effect of aged and fresh garlic extracts on fructosamine formation

**Figure 3:**
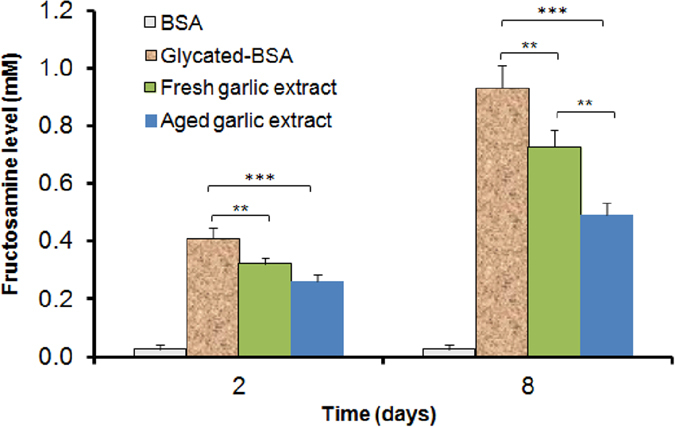
Effects of 10 mg/mL aged and fresh garlic extracts on fructosamine formation. BSA (10 mg/mL) was glycated by 0.5 M glucose in 0.1 M phosphate buffer, pH 7.4 for 10 days at 37 °C. Each value represents the mean ± SD (n = 3). At each time interval, statistical significance was defined as **P < 0.01, ***P < 0.001 compared to glycated BSA.

An increase in fructosamine occurs following incubation of BSA with glucose but not for BSA incubated alone (Fig. 3). Both extracts inhibited fructosamine production after 2 and 8 days of incubation with aged garlic being more effective compared to fresh garlic extract (Fig. 3). Compared to the glycated BSA, aged garlic extract showed significant (*P* < 0.001) inhibition in fructosamine formation after 2 and 8 days. Similarly, fresh garlic extract showed a significant (*P* < 0.01) inhibition of fructosamine formation. Fructosamine formation was inhibited more by aged garlic extract than by fresh garlic extract on day 8 (*P* < 0.01) but not on day 2 (Fig. 3).

### Effect of aged and fresh garlic extracts on glycation-induced protein carbonyl and thiol group formation

Bovine serum albumin glycated for 4 days showed an increase in protein bound carbonyl groups compared to BSA alone as a negative control (Table 1). Both extracts inhibited formation of protein-bound carbonyl groups with aged garlic extract being more effective (Table 1). Compared to glycated BSA, aged garlic extract showed significant (*P* < 0.001) inhibition of carbonyl group formation. Similarly, fresh garlic extract showed significant (*P* < 0.01) inhibition of carbonyl group formation. Glycated BSA had a significantly (*P* < 0.001) lower thiol content compared to BSA alone. Aged garlic extract had a significant (*P* < 0.001) protective effect against oxidation of thiol groups and fresh garlic extract also showed a significant (*P* < 0.01) effect compared to glycated BSA. Aged garlic extract was significantly (*P* < 0.05) more effective than fresh garlic extract in the prevention of thiol group oxidation (Table 1).

### Antioxidant activities of aged and fresh garlic extracts

In the 2, 2-azino-bis 3–ethylbenzthiazoline–6–sulfonic acid or ABTS assay, both extracts displayed antioxidant activities, as they were able to scavenge the ABTS^**+**^. Aged garlic extract had a higher Trolox equivalent antioxidant capacity (TEAC) compared to fresh garlic extract (Table 2). There was no significant difference in TEAC between aged garlic extract and ascorbic acid. However, fresh garlic extract showed a significant (*P* < 0.001) difference in TEAC compared to ascorbic acid. Aged garlic extract had a protective effect and significantly (*P* < 0.001) reduced TEAC compared to fresh garlic extract. In the 1, 1-diphenyl-2-picryl-hydrazyl (DPPH) radical scavenging assay, ascorbic showed significant (*P* < 0.001) inhibition of DPPH radicals compared to aged and fresh garlic extracts. The effect of aged garlic extract was significantly (*P* < 0.001) different from fresh garlic extract. Indeed, aged garlic extract demonstrated a stronger DPPH scavenging ability than fresh garlic extract (Table 2).

### Metal chelation activities of aged and fresh garlic extracts

**Figure 4:**
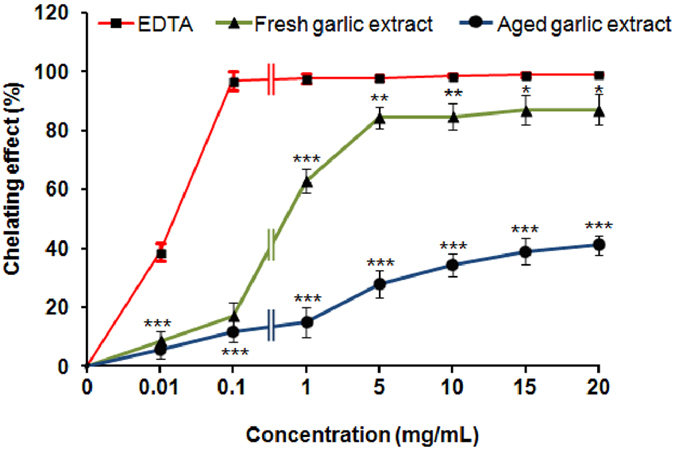
Effects of aged and fresh garlic extracts on metal chelating capacity. Each value represents the mean ± SD (n = 6). Statistical significance was defined as *P < 0.05, **P < 0.01, ***P < 0.001 compared to fresh garlic extract at each tested concentration.

Figure 4 showed the metal chelating effects of aged and fresh garlic extracts. Both extracts showed potent chelating abilities for ferrous ions. However, at all tested concentrations the fresh garlic had a stronger chelating effect compared to the aged garlic extract (Fig. 4). At higher concentrations (15–20 mg/mL), fresh garlic extracts showed significant (*P* < 0.05) chelating abilities for ferrous ions whereas aged garlic extract showed significant (*P* < 0.001) chelating effect compared to ethylenediaminetetraacetic acid (EDTA). However, EDTA showed significantly (*P* < 0.001) stronger Fe^2+^ -chelation activity even at minimal concentrations of 0.1 mg/mL compared to aged and fresh garlic extracts.

### Phenolic, flavonoid and flavonol compounds in aged and fresh garlic extracts

Table 3 shows the concentration of total phenolic compounds was significantly (*P* < 0.001) higher in aged garlic compared to fresh garlic extract. Similarly, the total flavonoid and flavonol concentrations of aged garlic were significantly (*P* < 0.001) higher than fresh garlic extract (Table 3).

## Discussion

There is considerable interest in antiglycation agents because of their therapeutic potential in reducing the morbidity and mortality associated with diabetes. Different strategies have been proposed to reverse the effects of AGEs, focusing on inhibition of AGE formation, removal of AGEs or interference with cellular effects of AGEs^2^. This study showed both aged and fresh garlic extracts inhibit the formation of AGEs and is in agreement with previous studies demonstrating aged garlic extract and SAC effectively inhibit AGE formation *in vitro*^12^. Indeed, aged garlic extract was shown to have more potent antiglycation properties compared to fresh garlic extract. Moreover, the addition of SAC to fresh garlic extract increased the inhibition of AGEs (results not shown). In addition to SAC, other compounds may be present in aged garlic extract contributing towards its antiglycation activity, although these compounds have not yet been determined. Fresh and aged garlic extracts inhibited early glycation products (fructosamine) formation possibly by modification of carbonyl or amino groups in the glycation reaction.

Protein oxidation has a key role in AGE formation and together with glycation generate protein-bound carbonyl groups, which have been detected in human tissues^13^. Furthermore, reaction with dinitrophenylhydrazine (DNPH) is a standard method for detecting carbonyl groups and can be used to follow changes in protein oxidation^14^. In this study, free carbonyl groups were generated by treating BSA with a high concentration of methylglyoxal. Aged garlic extract was more potent than fresh garlic in inhibiting protein-bound carbonyl groups with the results being comparable to the positive control, aminoguanidine and consistent with previous studies for aminoguanidine^14^.

Another protein modification that may potentially lead to structural changes is the loss of protein thiol groups. The oxidation of thiol groups was observed following glycation of BSA and this was inhibited by aged garlic extract and protects against oxidative protein damage. Therefore, the thiol assay provides mechanistic information concerning the effects of aged and fresh garlic extracts on AGEs.

Natural compounds with antioxidant properties can inhibit AGE formation. In this regard, compounds such as rutin^15^, garcinol^16^, loubuma tea^17^ and green tea^18^ have been shown to prevent AGE formation both *in vitro* and *in vivo.* Thus, the antiglycation activity of plant extracts could be attributed to their antioxidant properties. Indeed, several studies have demonstrated that the protective effects of garlic are due to their antioxidant and radical scavenging capacities^19^,^20^. The abilities of aged and fresh garlic extracts to scavenge ABTS**˙** were compared with ascorbic acid, the latter being an excellent marker for determining the antioxidant capacity of hydrogen donating antioxidants. The higher scavenging activity of aged garlic compared to fresh garlic extract was deemed similar to the antioxidant capacity of ascorbic acid and may be related to the high polyphenolic content of aged garlic extract. Antioxidant compounds can also reduce the purple colour of DPPH**˙** by providing hydrogen atoms or by electron donation and producing a colourless complex causing decreased absorbance, which is a measure of the extent of radical scavenging. The DPPH**˙** scavenging activity of phenolic compounds in aged and fresh garlic extracts is mainly due to their redox properties and depends on the chemical structure of the compound, the number of hydroxyl groups, the substitution pattern of hydroxyl groups and on the method of analysis^21^.

In the metal chelation assay, ferrozine forms complexes with ferrous ions and, in the presence of ferrous chelating agents; the complex formation is disrupted, resulting in reduction of the red complex colour. Therefore, measurement of colour reduction reflects metal chelation activity of coexisting chelators^22^. Aged and fresh garlic extracts interfere with the formation of ferrous and ferrozine complexes, therefore are able to capture ferrous ions before formation of ferrozine and accordingly produce metal chelation activity. Unexpectedly, fresh garlic extract produced stronger ferrous chelation activity compared to aged garlic extract, although the latter had higher flavonoid content. The metal-chelating ability of aged and fresh garlic extracts depend on their phenolic structure and the number and position of hydroxyl groups. It may be that the flavonoids in aged garlic extract do not have the required structure, accounting for this result. In addition, it is believed that acidic and/or basic amino acids may play an important role in the chelation of metal ions.

Aged garlic extract, which contains the highest amount of phenolic, flavonoid and flavonol compounds, exhibited the greatest antioxidant activity. These results were in agreement with those of previous workers who reported that the total phenolic content of aged garlic extract was higher than that of fresh and steamed garlic extracts^23^. Flavonoids and flavonols are important natural phenolics, and are the most widespread group of natural compounds. The mechanism(s) of action of flavonoids and flavonols are through the scavenging or chelating process^24^. Findings of this study support the hypothesis that phenolic compound in certain plant extracts effectively inhibit the formation of AGEs. Indeed, compounds with phenolic, flavonoid and flavonols content effectively inhibited the formation of dicarbonyl compounds and AGEs^25^. Therefore, the difference in antioxidant and antiglycation activities between aged and fresh garlic extracts could be due to their composition of phenolic and/or other non-phenolic compounds.

The aging of garlic converts the harsh, unstable and irritating compounds found in raw garlic such as allicin to stable unique and beneficial compounds. Thus, the processing method of aging garlic may influence its antioxidant activities. The high antioxidant activities of aged garlic extract might be due to antioxidant compounds other than phenolic compounds. The antiglycation activity of aged garlic extract is mainly due to its organosulphur compounds. Aged garlic extract contains mostly stable water-soluble organosulphur compounds such as SAC and SAMC, which are powerful antioxidants and are largely responsible for aged garlic extract’s health benefits. The pharmacokinetics of SAC is well established *in vivo*, which has high bioavailability and is used for standardizing aged garlic extract^26^. Both phenolic and organosulphur compounds present in garlic possess radical scavenging properties^27^. However, the presence of other potential antioxidant compounds, such as vitamin C, vitamin E and β-carotene, may also play an important role^21^. This antioxidant property of aged garlic extract may at least in part, account for its antiglycation properties.

This study has several limitations, one of which is the use of high sugar concentrations to speed up the glycation reaction, which otherwise occurs slowly *in vivo* being dependent on degree and duration of glycaemia. However, such an approach is useful for mechanistic studies allowing them to be conducted in an acceptable timescale and reducing the possibility of microbial sample contamination. It is unclear how these extracts would be metabolized *in vivo* and whether the relevant active ingredients would be present in blood at appropriate concentrations or whether any synergistic effects would still be operational. However, the approach used does provide a simple screening process to assess antiglycation and antioxidant properties of interesting extracts.

Dietary supplementation with antioxidants may be a safe and simple way of complementing traditional therapies aimed at targeting and preventing diabetic complications. This study demonstrates that aged and fresh garlic extracts protect against protein glycation *in vitro*. Aged garlic extract inhibits the formation of AGEs more effectively than fresh garlic extract, and this finding together with previous data suggest that daily consumption of aged garlic extract might be beneficial for prevention of lifestyle-related diseases. It is important to identify all compounds in aged garlic extract and to clarify their precise role. The bioavailability of different garlic components and their ability to inhibit AGE formation require further investigation. Further research is needed to clarify whether aged garlic extract attenuates the development of diabetic vascular lesions *in vivo*.

## Materials and Methods

Ethylenediaminetetraacetic acid was obtained from Fisher Scientific International, UK. Aged garlic extract was kindly provided by Wakunaga of America Company Ltd. Ribose, sodium dodecyl sulphate (SDS) and Tris (hydroxymethyl)-amino methane were obtained from BDH, UK. Acrylamide solution was obtained from Bio-Rad Laboratories (Hemel Hempstead, UK). Bromophenol blue was obtained from Serva, Germany. Ethanol, glacial acetic acid and methanol were obtained from Fisher Scientific International, UK. Dialysis tubing with a molecular weight cut off of 3.5 kDa and 67 kDa was obtained from Medical International Ltd Company (London, UK). Advanced glycation endproduct antibody (rabbit polyclonal to AGE antibody) and rabbit IgG secondary antibody (goat polyclonal to rabbit IgG) were purchased from Abcam, UK. All other reagents were of analytical reagent grade from Sigma-Aldrich Company (Poole, UK).

### Protein glycation

Briefly, either BSA or lysozyme (10 mg/mL) were incubated in 0.5 M glucose or 0.1 M methylglyoxal with or without prospective inhibitors (aged and fresh garlic extracts) in 0.1 M sodium phosphate buffer containing 3 mM sodium azide, pH 7.4 at 37 °C in the dark for a defined period of time^12^. Control samples with or without prospective inhibitors were incubated under the same conditions. Aliquots were stored at −20 °C until analysis.

### Preparation of fresh garlic extract

Fresh garlic was purchased from a retail food store (Manchester, UK). Garlic bulbs were identified by a botanist at the university. The bulbs (50 g) in good physical shape were peeled and weighed. Aqueous garlic extract was prepared according to an established method^28^. Briefly, 50 g of garlic was homogenized in 75 mL of cold 0.9% NaCl in the presence of some crushed ice. Homogenization was carried out in a blender at the highest speed with 1 minute bursts for a total of 12 minutes. The homogenized mixture was filtered through cheese cloth, centrifuged at 2000 × g for 10 minutes and the clear supernatant made up to 100 mL with saline. This aqueous garlic extract was stored in small aliquots at −20 °C until use. The dose of fresh garlic was calculated on the basis of the weight of fresh garlic in mg used to prepare a 1 mL extract (one millilitre of aqueous extract contained material from 500 mg of garlic). The aqueous garlic extract contains about 50% garlic material.

### Interrupted post-Amadori assay

Ribose-glycated protein rich in Amadori adducts was prepared as described previously^12^. Briefly, BSA (10 mg/mL) was incubated in 0.5 M ribose in 0.1 M sodium phosphate buffer containing 3 mM sodium azide, pH 7.4 at 37 °C for 24-hours. Unbound sugar was removed by extensive dialysis against distilled water and this Amadori-rich protein reincubated with or without the prospective extract at 37 °C. Aliquots were taken at various intervals (0–8 days) and frozen for subsequent analysis before measurement of AGEs using the enzyme-linked immunosorbent assay (ELISA) below.

### Measurement of fructosamine

Fructosamine content was quantified by a colorimetric method using nitroblue tetrazolium (NBT) as described previously^29^ and method calibrated using 1-deoxy-1-morpholino-D-fructose (DMF) standards.

### Measurement of AGEs

#### Crosslinked AGEs

These were assessed by the percentage crosslinking of the protein following sodium dodecyl sulphate-polyacrylamide gel electrophoresis (SDS-PAGE) using 10% gels as described previously^12^. These gels were stained with Coomassie blue, destained, photographed and analyzed using Gene snap programme from G Box Chem HR16 and Gene tool image analysis software from Bio Tools, Inc. (Edmonton, Alberta, Canada), respectively. Integrated density (I.D) was measured to analyze the one-dimensional electrophoretic gels and percentage inhibition of cross-linked AGEs was calculated using the formula:


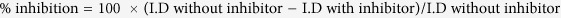


#### AGEs by ELISA

Microtiter plates were coated with 100 μL of standards (1 ng/mL-1 mg/mL) and samples (1 μg/mL) diluted in 50 mM carbonate buffer, pH 9.7, overnight at 4 °C. After coating, the wells were washed with 300 μL saline solution containing 0.05% Tween-20. The wells were treated with 200 μL of blocking buffer (3–5% non-fat dry milk or 5% serum BSA) in sodium carbonate buffer or PBS for 2 hours at room temperature, followed by washing. A volume of 100 μL of AGE antibody (rabbit polyclonal to AGE antibodies) as primary antibody was added at a suitable titre (1:2500 to 1:20000) diluted in blocking buffer and incubated for 2 hours at 37 °C, followed by washing. Then, 100 μL of rabbit IgG antibody; goat polyclonal to rabbit IgG (horse radish peroxidase) as the secondary antibody was added at a titre of 1:4000 to 1:10000 diluted in blocking buffer immediately before use and incubated for 1 hour at 37 °C, followed by washing. ABTS diammonium salt substrate solution was added (100 μL/well) to the plates followed by an incubation at room temperature for up to 30 minutes. A 100 μL of stop solution (0.625 M oxalic acid) was added, mixed and absorbance determined at 450 nm.

### Measurement of protein-bound carbonyl groups

Protein-bound carbonyl groups were measured using DNPH derivatisation as described previously^30^. The carbonyl content was calculated using a molar absorbance of 22,000 M^−1^cm^−1^ and the results expressed as the ratio of nmoles of DNPH reacted per mg of protein.

### Measurement of thiol groups

Free thiol content was determined in BSA alone and in glycated BSA in the presence or absence of extracts according to an established method^31^ using 5,5′-dithiobisnitrobenzoic acid (DTNB) and L-cysteine hydrochloride as standards. Free thiol concentration was calculated using 14,150 M^−1^cm^−1^ as the molar extinction coefficient of DTNB. The results were expressed as the ratio of nmoles of DTNB reacted per mg of protein.

### ABTS antioxidant assay

The standard ABTS assay^32^ was used for determination of TEAC of both aged and fresh garlic extracts. Trolox dissolved in ethanol was used as a reference standard (0–20 μM) whereas ascorbic acid was used as a positive control.

### DPPH radical scavenging capacity assay

1, 1-Diphenyl-2-picryl-hydrazyl radical scavenging activity of extracts was measured according to a modified version of an existing method^33^. Trolox was used as the reference standard compound and ascorbic acid was used as positive control. Antioxidant activity of a sample was expressed in terms of micromole equivalents of trolox. The concentration causing 50% inhibition (IC_50_) of each extract was determined and compared with the corresponding TEAC.

### Metal chelation assay

The ability of garlic extracts to chelate ferrous ions (Fe^2+^) was assessed according to a modified version of an established method^34^. EDTA was used as a positive control and diluted to 0.01–20 mg/mL for further use. The chelating activity of the extract for Fe^2+^ was calculated using the formula:


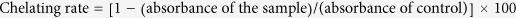


### Measurement of total phenolic compounds

The amount of total phenolic compounds in garlic extracts was estimated according to a modified version of a previously reported method using Folin-Ciocalteu reagent^35^. The method was calibrated using 0.024–0.3 mg/mL ethanolic gallic acid solution (10 g/L) and results were expressed as milligrams per gram of extract of gallic acid equivalents.

### Measurement of total flavonoids

The flavonoid content was determined according to a modified version of a previously described colorimetric procedure using quercetin as a reference compound^36^. Quercetin was used for standards and the flavonoid content was expressed in milligrams of quercetin equivalent per gram of extract.

### Measurement of total flavonols

The content of flavonols was determined according to an established method^35^. Rutin standards were used to calibrate the method and flavonol content was expressed as milligrams of rutin equivalent per gram of extract.

### Statistical analysis

Statistical analysis was performed using SPSS 19 software (SPSS Inc., Chicago, Illinois, USA). Data were presented as mean ± standard deviation (SD) from at least three independent experiments. The significant difference between test groups was analyzed using two-way analysis of variance (2-factor ANOVA), followed by Bonferroni Post Hoc tests for the comparisons between tested concentrations in aged and fresh garlic extracts. A Dunnett’s Post Hoc test was used to compare between aged and fresh garlic extracts against the control. Statistical significance was defined as *P < 0.05, **P < 0.01, ***P < 0.001.

## Additional Information

**How to cite this article**: Elosta, A. *et al*. Aged garlic has more potent antiglycation and antioxidant properties compared to fresh garlic extract *in vitro. Sci. Rep.* **7**, 39613; doi: 10.1038/srep39613 (2017).

**Publisher's note:** Springer Nature remains neutral with regard to jurisdictional claims in published maps and institutional affiliations.

## Figures and Tables

**Table 1:** Effects of 10 mg/mL aged and fresh garlic extracts on protein carbonyl and thiol groups of methylglyoxal- and glucose-modified BSA respectively.

	Carbonyl groups nmol/mg protein	Thiol groups pmol/mg protein
BSA	0.4 ± 0.2	11.4 ± 0.9
Glycated BSA in absence of extracts	12^C^ ± 0.4	5^C^ ± 0.2
Glycated BSA in presence of aged garlic extract	5.9^Ab^ ± 0.5	9.4^Ab^ ± 0.5
Glycated BSA in presence of fresh garlic extract	8.3^Ba^ ± 0.6	8^Ba^ ± 0.5

Data are presented as mean ± SD (n = 6). The symbols ^A^ and ^B^ refer to the significant difference between labelled values as compared to the glycated BSA in the absence of extracts. Labelled means in each column without common uppercase letters differ significantly from glycated-BSA, *P* < 0.001 and *P* < 0.01 respectively. Labelled means in each column without common lowercase letters differ significantly from aged garlic extract, *P* < 0.05.

**Table 2:** Antioxidant activities of ascorbic acid, aged garlic and fresh garlic extracts as determined by ABTS and DPPH radical scavenging assays.

	ABTS [TEAC] (μM Trolox)	DPPH [IC_50_] (mg/mL)
Ascorbic acid	19.5^C^ ± 0.3	11.7^C^ ± 0.2
Aged garlic extract	19^Cb^ ± 0.1	15^Ab^ ± 0.2
Fresh garlic extract	12^Aa^ ± 0.7	24^Ba^ ± 0.5

Data are presented as mean ± SD (n = 6). The symbol ^A^ and ^B^ refer to the significant difference between the labelled values as compared to the ascorbic acid. Labelled means in each column without common uppercase letters differ significantly from ascorbic acid, *P* < 0.001. Labelled means in each column without common lowercase letters differ significantly from aged garlic extract, *P* < 0.001.

**Table 3:** Total amount of phenolic, flavonoid and flavonol compounds in aged and fresh garlic extracts.

	Total phenolics mg/g	Total flavonoids mg/g	Total flavonols mg/g
Aged garlic extract	129^A^ ± 1.8	101^A^ ± 1.8	94^A^ ± 0.9
Fresh garlic extract	56^B^ ± 1.2	47^B^ ± 6	43^B^ ± 3.3

Data are presented as mean ± SD (n = 6). The symbol ^A^ refers to the significant difference between the labelled values as compared to the aged garlic extract. Labelled means in each column without common uppercase letters differ significantly from aged garlic extract, *P* < 0.001.
